# Electrophysiological Properties and Viability of Neonatal Rat Ventricular Myocyte Cultures with Inducible ChR2 Expression

**DOI:** 10.1038/s41598-017-01723-2

**Published:** 2017-05-08

**Authors:** Qince Li, Rong (Ruby) Ni, Huixian Hong, Kah Yong Goh, Michael Rossi, Vladimir G. Fast, Lufang Zhou

**Affiliations:** 1 Department of Medicine, Division of Cardiovascular Disease, University of Alabama at Birmingham, 1900 University Blvd, Birmingham, 35294 Alabama USA; 2 Department of Biomedical Engineering, University of Alabama at Birmingham, 1670 University Blvd, Birmingham, 35294 Alabama USA; 3 Cardiac Rhythm Management Laboratory, University of Alabama at Birmingham, Birmingham, 35294 Alabama USA; 4 Comprehensive Cardiovascular Center, University of Alabama at Birmingham, Birmingham, 35294 Alabama USA

## Abstract

Channelrhodopsin-2 (ChR2)-based optogenetic technique has been increasingly applied to cardiovascular research. However, the potential effects of ChR2 protein overexpression on cardiomyocytes are not completely understood. The present work aimed to examine how the doxycycline-inducible lentiviral-mediated ChR2 expression may affect cell viability and electrophysiological property of neonatal rat ventricular myocyte (NRVM) cultures. Primary NVRMs were infected with lentivirus containing ChR2 or YFP gene and subjected to cytotoxicity analysis. ChR2-expressing cultures were then paced electrically or optically with a blue light-emitting diode, with activation spread recorded simultaneously using optical mapping. Results showed that ChR2 could be readily transduced to NRVMs by the doxycycline-inducible lentiviral system; however, high-level ChR2 (but not YFP) expression was associated with substantial cytotoxicity, which hindered optical pacing. Application of bromodeoxyuridine significantly reduced cell damage, allowing stimulation with light. Simultaneous optical V_m_ mapping showed that conduction velocity, action potential duration, and dV_m_/dt_max_ were similar in ChR2-expressing and control cultures. Finally, the ChR2-expressing cultures could be optically paced at multiple sites, with significantly reduced overall activation time. In summary, we demonstrated that inducible lentiviral-mediated ChR2 overexpression might cause cytotoxicity in NRVM cultures, which could be alleviated without impairing electrophysiological function, allowing simultaneous optical pacing and V_m_ mapping.

## Introduction

The optogenetic technology, which allows remote control of electrical activity of cells of interest with high spatiotemporal resolution using light, has experienced explosive growth in basic and translational neuroscience in the last decade^1–7^. Recently, this innovative technique has been introduced to cardiac research both *in vitro* and *in vivo* to trigger^8–14^ or terminate electrical activity of cardiomyocytes^15^. For instance, Jia *et al*. described a “tandem cell unit” cell culture model that consisted of neonatal rat ventricular myocytes (NRVMs) co-cultured with HEK cells carrying the light-gated Channelrdoposin 2 (ChR2) gene^10^. They showed that illumination of the HEK cells led to depolarization and electrical excitation of NRVMs. Similarly, Nussinovitch *et al*. showed that optical illumination of fibroblasts expressing ChR2 or Archaerhodopsin-T can be used to stimulate or inhibit the electrical activity of co-cultured NRVMs^11, 16^. Those work proved the feasibility of indirect optogenetic control of cardiomyocytes in the dish. In addition to those *in vitro* studies, several animal studies have demonstrated that direct optogenetic cardiac pacing can be performed *in vivo* or *ex vivo* on transgenic mice^8^, zebrafish^9^ and rats^17^. Given those advancements, the application of optogenetic techniques in the treatment and diagnosis of cardiac diseases is still at the infant stage, partially due to the lack of well-characterized cardiac optogenetic models that contain the genetic encoded light-sensitive proteins. While transgenic animals are valuable and widely used for basic mechanistic studies, translational and clinical application of cardiac optogenetics would rather require using alternative methods for genetic manipulation, e.g., viral vector-based delivery of optogenetic constructs into cells or tissue of non-transgenic diseased hearts.

Lentiviruses have been extensively used as gene delivery vectors since the mid-1990s. Due to their ability to integrate into the genome of target cells, lentiviruses can infect both dividing and non-dividing cells and confer stable high-level expression^18^. This characteristic renders them of vital interest for gene therapy of cancer, cardiovascular disease, infectious disease, and neurodegenerative diseases. In optogenetic research, lentiviruses have achieved stable and effective rhodopsin proteins (e.g. ChR2) expression in a variety type of cells including neurons^2^, skeletal muscle cells^19^, and stem cells^20^. Recently, the use of lentiviruses for ChR2 delivery has been extended to cardiac optogenetic research, including neonatal cardiomyocytes^21^ and human pluripotent stem cells derived cardiomyocytes^20, 22^. An important question before cardiac optogenetic technique can be translated to clinical application is whether, and if yes, how the lentiviral-induced ChR2 expression affects cardiomyocyte viability and electrophysiological property. Viral-mediated ChR2 overexpression has been shown to induce cytotoxicity in neurons and glial cells^23–25^. In cardiac cells, however, this aspect has not yet been fully evaluated. In addition, the application of inducible optogenetic (ChR2) gene expression technique, which could benefit long-term gene therapy^26^, in cardiac optogenetics has not been examined.

Another critical element of cardiac optogenetic application is simultaneous and accurate recording of activation spread initiated by light stimulation. Optical mapping is a widely-used experimental technique for monitoring cardiac excitation^27^, which employs fluorescent dyes to image membrane potential (V_m_) and other parameters such as intracellular Ca^2+^ transients at high spatial and temporal resolutions^28–30^. Since its introduction, optical mapping has revolutionized the study of impulse conduction and cardiac rhythm. Therefore, optical mapping could be a method of choice in cardiac optogenetics studies. However, as both methods employ visible light, their integration represents a challenge. Only very recently, Park *et al*. reported the first study of simultaneous optical control and V_m_ recordings in cardiomyocyte cultures^21^. However, their work primarily focused on the light-induced modulation of action potential duration (APD).

To address those issues, we developed and characterized an optogenetic NRVM culture model containing doxycycline (Doxy)-inducible lentiviral-mediated ChR2 expression. The results showed that the lentivirus-induced ChR2 protein overexpression caused substantial cytotoxicity in NRVM cultures, which could be alleviated by the application of bromodeoxyuridine (BrdU), allowing direct optical pacing and simultaneous V_m_ optical mapping. We also demonstrated that moderate ChR2 expression and light pacing did not influence the basic electrophysiological property of NRVMs. Preliminary results of this work have been published in abstract form^31^.

## Results

### Effects of viral transduction and ChR2 expression on NRVM cultures

**Figure 1:**
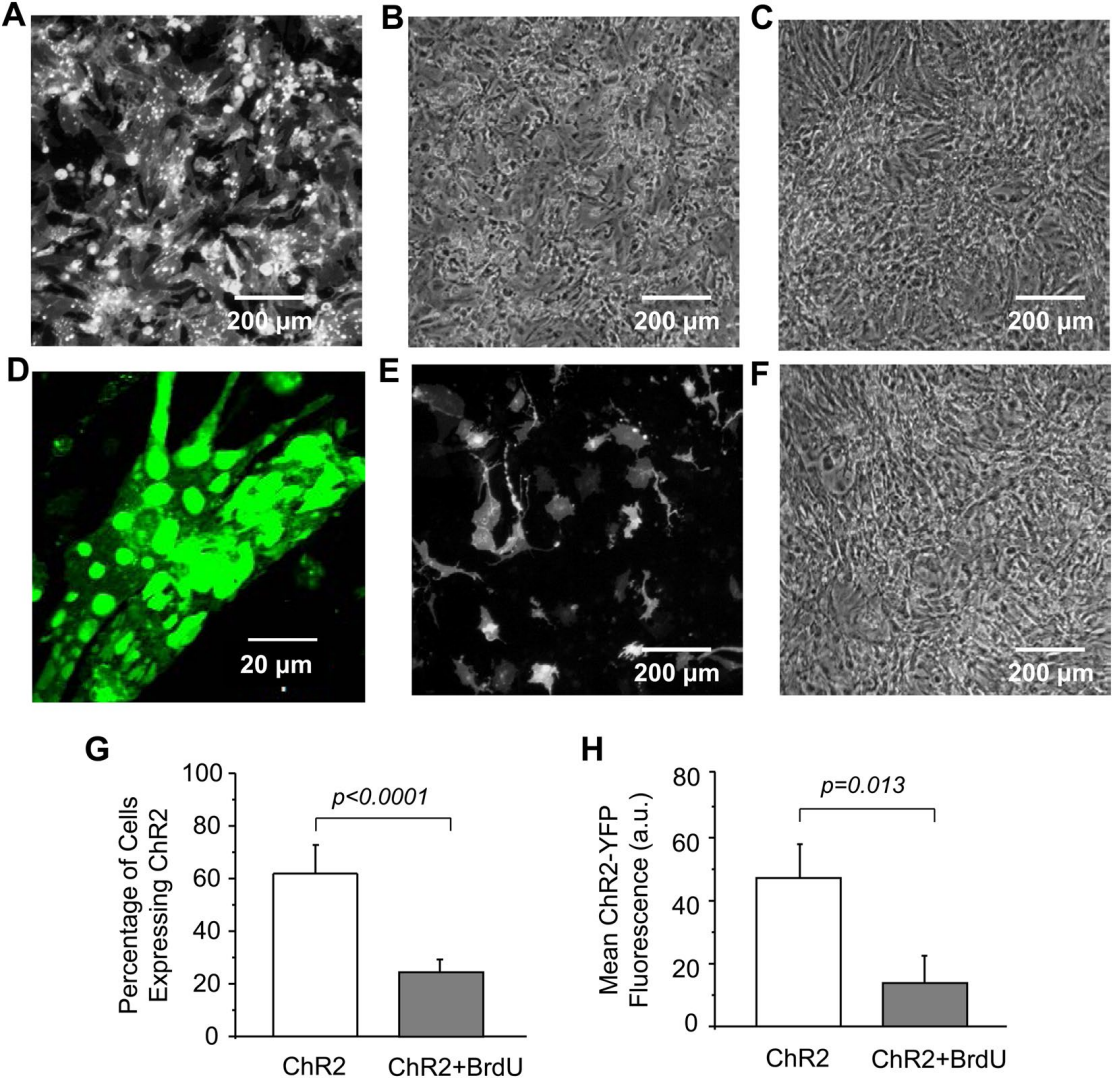
Effects of lentivirus transduction and doxycycline-induced ChR2 expression on NRVM cultures. (**A** and **B**) Fluorescent and phase-contrast images, respectively, of a ChR2-transduced monolayer; (**C**) Phase-contrast image of a control monolayer; (**D**) Confocal image of ChR2-expressing cardiomyocytes; (**E** and **F**) Fluorescent and phase-contrast images, respectively, of a ChR2-expressing monolayer pretreated with BrdU; (**G**) ChR2 transduction efficiency in cell cultures with or without BrdU pretreatment (two-tailed unpaired Student’s t test, n = 6); and (**H**) Effect of BrdU on ChR2-eYFP fluorescence intensity averaged over monolayer (two-tailed unpaired Student’s t test, n = 6).

We first examined the Doxy-activated lentivirus-mediated ChR2 expression in NVRMs and its effect on cell culture viability and electrical property. When the multiplicity of infection (MOI) was small (e.g. 10 or 20), the transduction efficiency (i.e. the percentage of ChR2-expressing positive cells) and the overall level of ChR2 expression were low; those monolayers could barely respond to (470 nm) LED light stimulation. Increasing MOI (e.g. to 40) led to high-level, homogeneous ChR2 expression throughout cell monolayer (Fig. 1A). However, severe cell damage was found in those cultures, including significantly lower (40.1 ± 4.0%, n = 10) cell density (Fig. 1B) as compared to controls (Fig. 1C) and adverse morphological changes such as smaller cell size, membrane blebbing, and strong ChR2 protein aggregation in the cytoplasm (Fig. 1D). Importantly, such monolayers usually developed rapid spontaneous beating and were unresponsive to external (optical or electrical) stimulation.

To solve these problems, NRVM monolayers were treated with BrdU, a DNA replication inhibitor, prior to viral transduction. BrdU pretreatment led to a reduced and less uniform ChR2 expression (Fig. 1E) but alleviated cell damage. The cell morphology and density in the BrdU-treated ChR2 NRVM cultures (Fig. 1F) were similar to that in the control cultures (Fig. 1C). BrdU had no significant effect on the non-infected cell cultures (data not shown), but significantly reduced the viral transduction efficiency (Fig. 1G) and overall ChR2 expression (Fig. 1H) as compared to the non-treated cultures.

**Figure 2:**
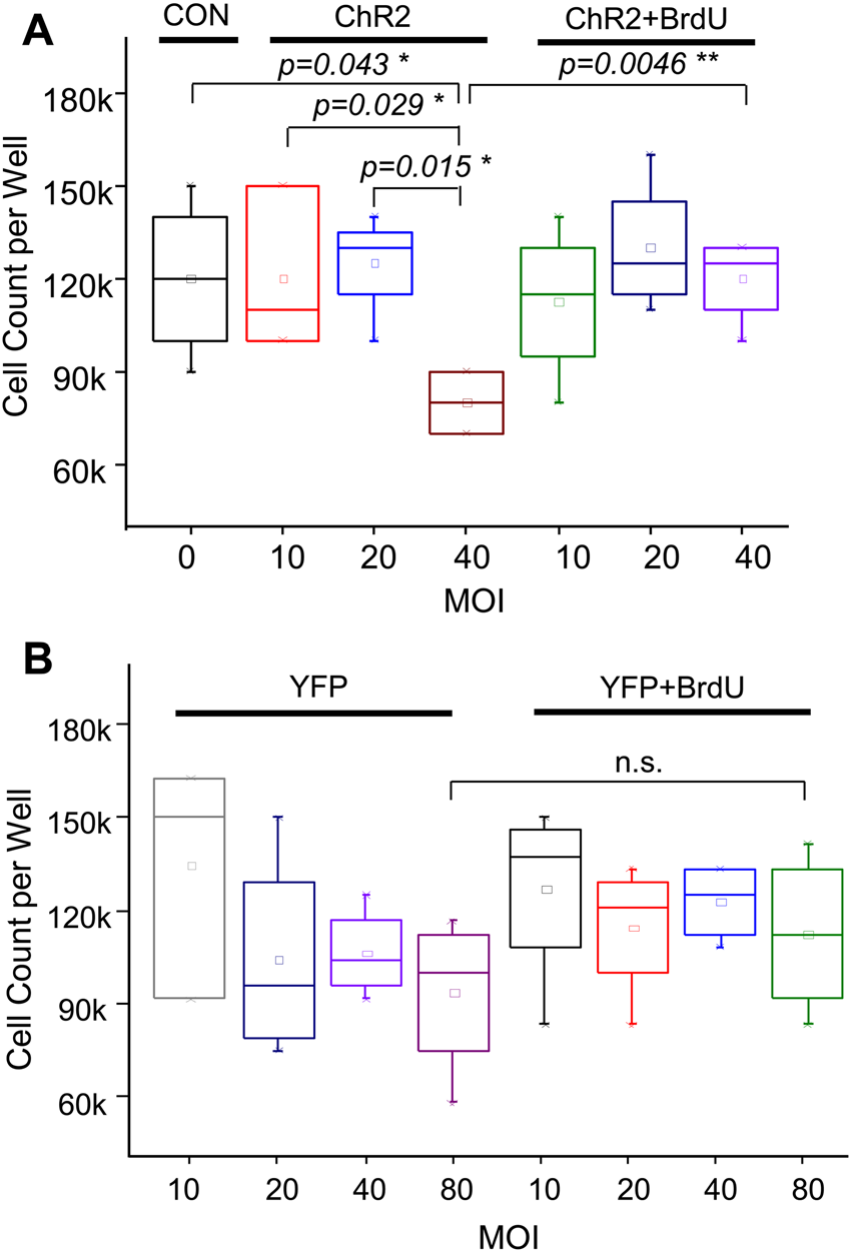
Effects of infection MOI and BrdU treatment on NRVM viability in ChR2 (**A**) or YFP (**B**) lentivirus-infected cultures. *: one-way ANOVA, **: two-way ANOVA, n = 3 or 4 per group.

To examine the cause of cytotoxic effects observed in the ChR2-transduced cardiomyocytes, the gene transduction procedure was varied and cell viability was quantified using the trypan blue assay. Procedure modifications included application of ChR2 lentivirus at different MOIs, use of different virus batches, treatment with or without BrdU and Doxy, and use of YFP-only virus. Data showed that when the MOI was relatively low (e.g., 10 and 20) the cell viability of the ChR2-transduced cultures was similar to that of non-infected cultures (i.e. MOI = 0), regardless of whether cells were pretreated with BrdU or not (Fig. 2A). However, when the MOI was increased to 40, the cell viability declined dramatically in the ChR2-infected NRVM monolayers that were not treated with BrdU as compared with the control and low MOI-transduced cultures (Fig. 2A). BrdU pretreatment substantially reduced the high MOI infection-induced cell damage; the number of cells per well was not significantly different from that of control cultures treated with Doxy and BrdU only (Fig. 2A). The similar effects of MOI and BrdU treatment on ChR2-expressing cardiomyocytes have been observed in NRVM cultures infected with various batches of ChR2 lentivirus (see Figure S2 for an example). It is worth mentioning that neither BrdU nor Doxy treatment alone had significant effect on cell viability (Figure S3).

To examine whether the cytotoxicity was caused by the viral-mediated ChR2 expression or the viral infection itself, NRVM cultures were infected with YFP-only lentivirus with various MOIs. Cell viability analysis shows that YFP lentiviral infection (and the mediated YFP expression) had no detrimental effect on NRVM cultures, regardless of the MOI or the BrdU treatment (Fig. 2B). In particular, no substantial cytotoxicity was detected in monolayers infected with YFP-only lentivirus, even with high MOIs (e.g. 80) and without the presence of BrdU. The effects of BrdU treatment on cell viability in YFP- or ChR2-transduced NRVM cultures with low (i.e. 10) and high (i.e. 40) MOIs are summarized in Figure S4.

**Figure 3:**
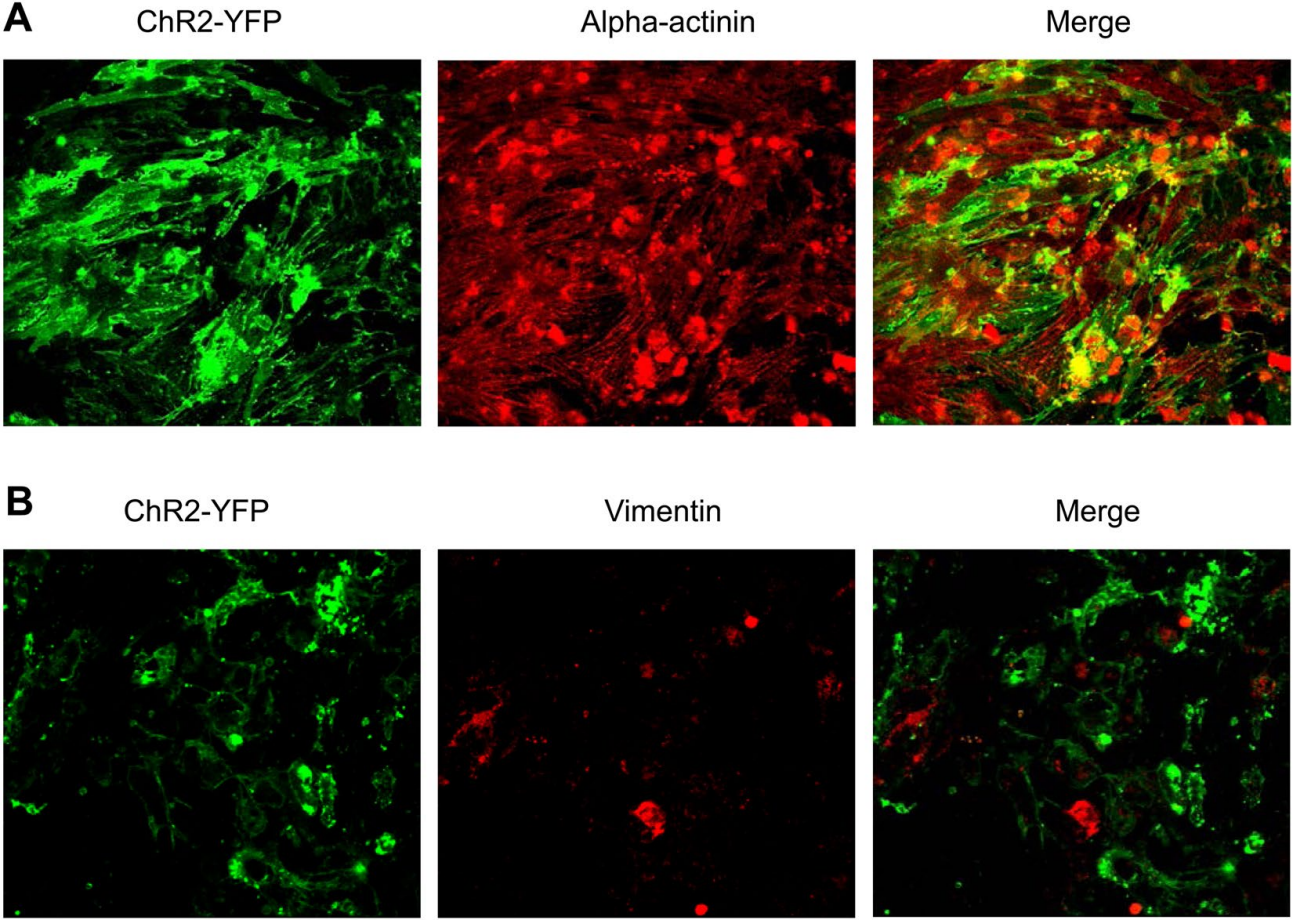
Characterization of lentivirus transduction-induced ChR2 expression in cardiomyocytes and fibroblasts. (**A**) NRVM culture stained with anti-sarcomeric alpha-actinin antibody; and (**B**) NRVM culture stained with anti-vimentin antibody. n = 4 per group.

Primary NRVM cultures often contain a certain number of non-myocyte cells, primarily fibroblasts^32^, which could be electrically coupled to cardiomyocytes^33^. If fibroblasts express ChR2, they may contribute to optogenetic stimulation of cardiac cells^10^. Therefore, we characterized the type of ChR2-expressing cells in the cultures using an anti-sarcomeric alpha-actinin antibody (a cardiomyocyte marker) and an anti-vimentin antibody (a fibroblast marker). The image analysis showed strong staining for alpha-actinin, which was largely overlapped with or in close proximity to ChR2-eYFP (Fig. 3A). On the contrary, NRVM cultures displayed much less staining for vimentin, which was barely colocalized with ChR2-eYFP (Fig. 3B). Qualitatively similar results were obtained in a total of eight cultures (n = 4 for each group). These results indicated that there were few fibroblasts in NRVM cultures and ChR2 was expressed mainly in cardiomyocytes.

### Direct optical pacing of ChR2-expressing NRVM cultures

**Figure 4:**
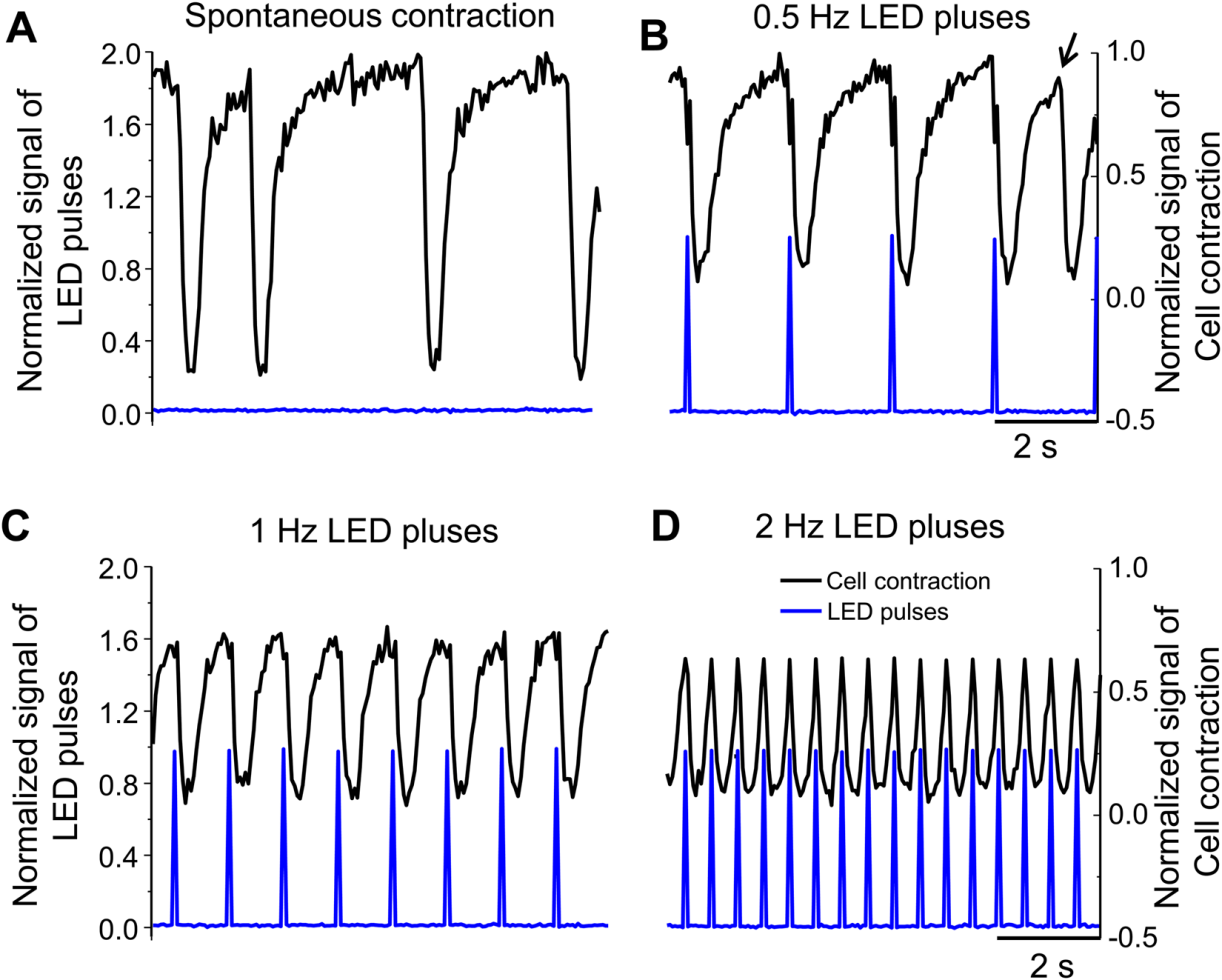
Direct optical pacing of ChR2-expressing NRVM cultures at varying light pulse frequencies. (**A**) Spontaneous contractions of a culture recorded in transmitted light; (**B**–**D**) Cell contractions triggered by light pacing at a frequency of 0.5, 1 and 2 Hz, respectively. The arrow marks an uncaptured contraction at the 0.5 Hz pacing rate.

We next examined whether BrdU treatment enables direct optical pacing of cardiomyocytes containing Doxy-inducible ChR2 expression (hereinafter refers to NRVM cultures infected by ChR2 lentivirus at MOI of 40 and in the presence of BrdU and Doxy). As shown in Fig. 4A and Movie S1 (supplemental information), NRVMs beat spontaneously and irregularly prior to optical stimulation at a rate of ~0.3 Hz. Optical pacing with blue LED light at faster rates resulted in rhythm capture. In our hands, the slowest rate of light stimulation was about 0.5 Hz (Fig. 4B) although at such low pacing frequency the rhythm capture was incompletely as extra spontaneous cell contractions could be frequently observed (Fig. 4B, marked by the arrow). Further increasing pacing frequency (e.g. to 1 and 2 Hz) led to complete capture of cell culture rhythm by LED pulses (Fig. 4C and D), demonstrating the feasibility of direct optogenetic pacing of NRVM cultures. The highest frequency of optical pacing resulting in stable rhythm capture was 3.1 Hz (Movie S1). Similar results were obtained in a total amount of five monolayers.

### Simultaneous optical pacing and V_m_ mapping of ChR2-expressing NRVM cultures

**Figure 5:**
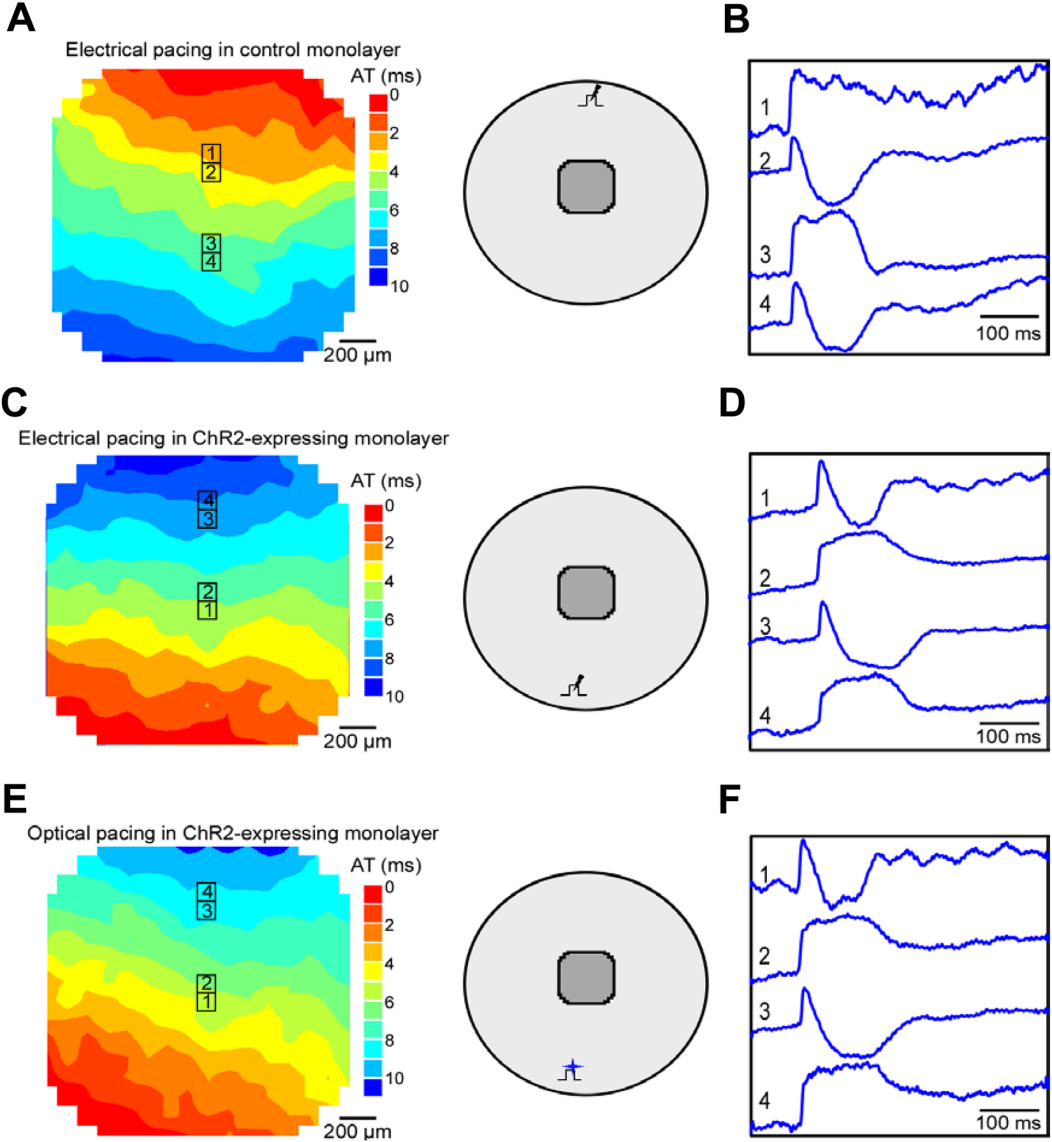
Isochronal map of activation spread and selected V_m_ traces in NRVM monolayers. (**A** and **B**) A control monolayer during electrical pacing; (**C** and **D**) A ChR2-expressing monolayer during electrical pacing; (**E** and **F**): A ChR2-expressing monolayer during optical pacing. The schematic insets show the corresponding pacing site and mapping area. The black lightning symbol in the inset represents electrical pacing and the blue star symbol represents optical stimulation.

Normal AP propagation is critical for maintaining cardiac function. To examine the properties of light-triggered AP propagation in ChR2-expressing NRVM cultures, optical V_m_ mapping was performed during optical or electrical pacing (2 Hz). We first examined the patterns of electrical stimulation-induced AP propagation in control and ChR2-expressing NRVM monolayers. Optical mapping showed that activation spread had uniform patterns in ChR2-expressing monolayers (Fig. 5C and D), which were similar to those in the control cultures (Fig. 5A and B). The same ChR2-expressing monolayers were then subjected to stimulation by optical pulses and the AP propagation were recorded. Data showed that the activation patterns elicited by optical pulses (Fig. 5E and F) were very similar to those induced by electrical stimulus (Fig. 5C and D).

**Figure 6:**
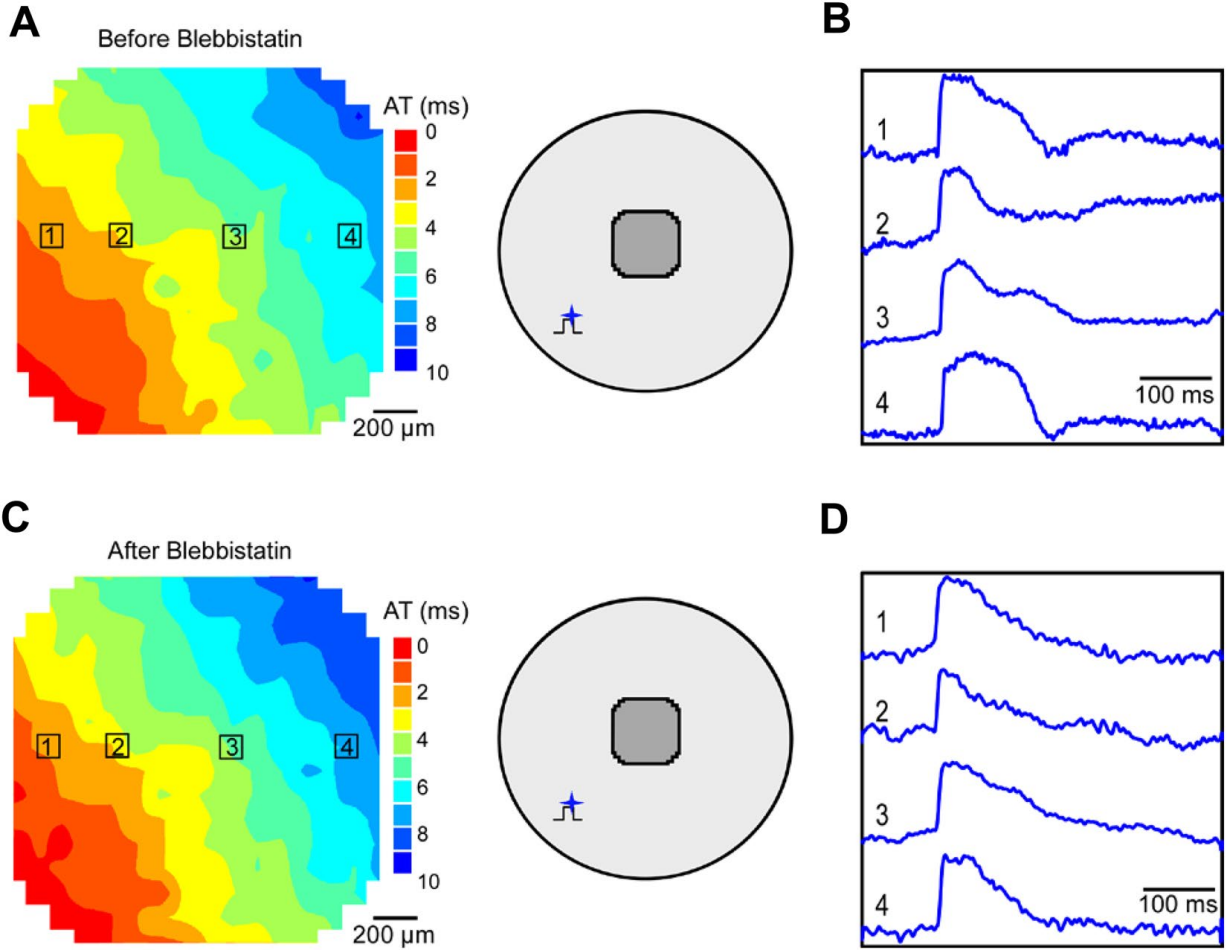
Suppression of motion artifact by blebbistatin. Activation maps and optical V_m_ recordings before (**A** and **B**) and after (**C** and **D**) blebbistatin application in a ChR2-expressing NRVM culture. The schematic insets show optical pacing site and mapping area.

We next measured the electrophysiological parameters of AP propagation (i.e., APD, dV/dt_max_, and conduction velocity) in the control and ChR2-expressing NRVM cultures paced electrically or ChR2-expressing NRVM cultures paced with light. Typically, APD measurements were hindered by cell motion artifact, which can be seen in Fig. 5 as deflections following AP upstrokes; such deflections often had opposite polarities at neighboring sites (e.g., sites 3 and 4 in Fig. 5A,C and E). Application of blebbistatin effectively suppressed motion artifact in AP recordings in control monolayers (Figure S5) and ChR2-expressing cell cultures (Figure S6) paced electrically. Blebbistatin had minimal effect on the activation map and conduction velocity in those monolayers (Figure S7A and B). Analysis of the V_m_ optical mapping data showed that the mean values of conduction velocity, APD_80_, and dV/dt_max_ were not significantly different between the ChR2-expressing (n = 6) and control cultures (n = 8) (Table 1), which is consistent with results reported by Park *et al*.^21^. Similarly, as in the monolayers paced electrically blebbistatin successfully suppressed the motion artifact but did not alter the light-induced AP propagation (Fig. 6) and conduction velocity (Figure S7C) in ChR2-expressing NRVM monolayers. In addition, there were no significant differences in the basic electrophysiological parameters in the ChR2-expressing cell cultures paced electrically or optically (Table 1), which are consistent with previous studies^13^.

**Table 1:** Electrophysiological parameters (conduction velocity, APD_80_ and dV/dt_ma_) measured in control and ChR2-expressing NRVM cultures.

	Conduction velocity (cm/s)	APD_80_ (ms)	dV/dt_max_ (V/s)
Control cultures Electrical pacing (n = 8)	19.3 ± 4.1	114.5 ± 29.8	32.3 ± 5.0
ChR2-expressing Electrical pacing (n = 8)	18.9 ± 1.0^*^ (p = 0.44)	124.7 ± 29.2^*^ (p = 0.12)	32.7 ± 2.9^*^ (p = 0.87)
ChR2-expressing Optical pacing (n = 6)	19.5 ± 1.4^#^ (p = 0.557)	133.8 ± 20.1^#^ (p = 0.152)	32.3 ± 6.5^#^ (p = 0.387)

Pacing frequency = 2 Hz, culture duration ranged from 6 to 8 days. *Non-significant *vs*. control cultures paced electrically (two-tailed unpaired Student’s t test). ^#^Non-significant *vs*. ChR2-expressing cultures paced electrically (two-tailed unpaired Student’s t test).

### Multisite optical pacing of NRVM cultures expressing ChR2

**Figure 7:**
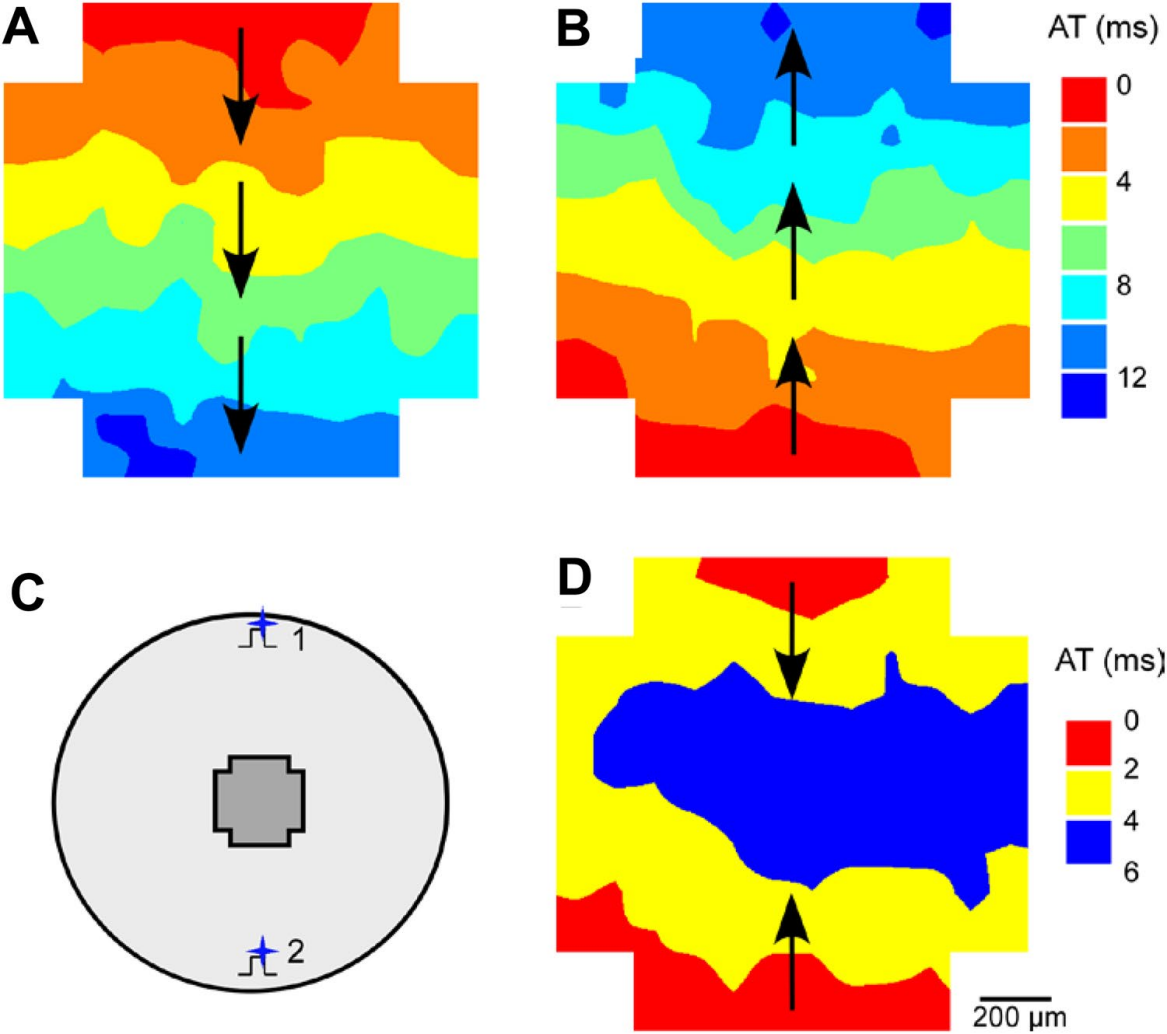
Multisite optical pacing of a ChR2-expressing NRVM culture. (**A** and **B**) Activation maps during pacing at a single site located on top or bottom of the cell culture, respectively; (**C**) A diagram schematically showing optical pacing sites and mapping area; (**D**) Activation maps of synchronous two-site pacing.

To examine the feasibility of multisite optogenetic pacing, we simultaneously illuminated a ChR2-expressing cell culture at two different sites and mapped the triggered AP propagation. Figure 7A and B show the AP propagation when the cell culture was paced optically at one site located either above or below the mapping area, respectively (sites 1 or 2 schematically shown in Fig. 7C). During single site optical pacing, the overall activation time across the mapping area was approximately 14 ms, which was not affected significantly by the location of pacing site. During dual-site pacing (Fig. 7D), two activation wavefronts were initiated at the top and bottom illumination sites simultaneously and travelled in the opposite direction colliding in the center of the monolayer. In this case, the overall activation time within the mapping area reduced to ~6 ms that is about half of that of single site pacing, suggesting that optogenetic pacing can be expanded to multiple sites.

## Discussion

In the present work, we developed and characterized an optogenetic NRVM culture model that is based on Doxy-inducible ChR2 expression. In particular, we examined the effect of lentivirus-mediated ChR2 expression on cell viability and electrophysiological property of NRVM cultures as well as the feasibility of direct optical pacing of ChR2-expressing cardiomyocytes. The main findings are: 1) infection of NRVM cultures with high MOI ChR2 lentivirus, but not YFP lentivirus, caused strong cardiomyocyte toxicity; 2) BrdU treatment alleviated cytotoxicity in ChR2-expressing cells; under such conditions, NRVM cultures could be stably paced using light pulses; and 3) optogenetic pacing had minor effects on the electrophysiological property of NRVM cultures.

Inducible gene expression system is a valuable tool in cardiac optogenetic research and translational application as it allows for temporally and spatially controlled activation of light-sensitive proteins. For instance, tetracycline inducible lentiviral system can be used in animal model systems by expressing rtTA in specific heart cells (such as Purkinje cells or atrial cardiomyocytes) to allow optogenetic manipulation of desired cell types. Our results showed that ChR2 genes were readily transduced into NRVMs using a Doxy-inducible lentiviral vector, rendering homogeneous and high-level ChR2 protein expression. However, severe cell damage and detrimental electrophysiological alterations were often found in those NRVM cultures, which hindered optical pacing and rhythm capture. Similar optogenetic protein expression-induced cell malformation and toxicity have been previously reported in neurons and glial cells^23–25^. The observed cytotoxicity could be a major concern in translational and clinical applications of cardiac optogenetics, but the underlying mechanism is not entirely clear. One possibility is that the cell damage was caused by the lentivirus infection, as primary cells such as cardiomyocytes may be sensitive to high titers. However, the fact that YFP-only lentivirus infection did not cause toxic effect to NRVM cultures, even with higher MOIs, indicated that this was not the case. In addition, we found that BrdU treatment substantially reduced cytotoxicity in the ChR2 lentivirus-infected cell cultures, providing further support that the cytotoxic effect was not caused by viral infection because lentivirus doesn’t proliferate and therefore BrdU shouldn’t reduce the number of viral particles. Our data also showed that BrdU or Doxy treatment alone had no effect on NRVM monolayers. Taken together, it seemed like that the observed cytotoxicity was mainly caused by the Doxy-induced ChR2 overexpression.

While the possible effect of BrdU on protein expression was barely investigated, a study by Rauth *et al*. showed that BrdU treatment caused a marked decrease in the level of tyrosinase protein and mRNA expression^34^. In a following study, they reported that suppression is not due to incorporation of BrdU into the coding sequence of the tyrosinase or the upstream tyrosinase gene sequences in the plasmids^35^. Thus BrdU may not suppress ChR2 gene directly but instead inhibit its activator or regulatory genes, as suggested by a previous study^36^. Apparently, the exact mechanism by which BrdU prevents Doxy-inducible ChR2 overexpression requires further investigation. One downside of BrdU treatment was weaker and less uniform ChR2 expression, which may compromise the capability for precise spatial control of cells with light. In addition, as BrdU can replace thymidine during DNA replication and cause mutations, using it *in vivo* should be very careful. For instance, it has been reported that BrdU caused cardiovascular malformations in the chick embryo^37^. We also noticed that rather long duration of optical pulses (e.g. 20 ms) was required for reliable rhythm capture. Although such pulse durations were comparable to those used in previous cardiac optogenetics studies^10, 11, 21^, they are significantly longer than the durations used in electrical pacing (2–5 ms). The longer pulses required for optical pacing can be due to (i) relatively low level of ChR2 expression and (ii) relatively long time constant of ChR2 channel activation, which could be as long as 5 ms^5, 38^. Indeed, we have found that when the ChR2 transduction efficiency is low (e.g. <10%), the infected monolayers could barely be optically paced.

The high-level ChR2 protein expression-mediated cytotoxicity could happen *via* several mechanisms. One possible mechanism is related to intracellular acidification and Ca^2+^ overload. Although the largest portion of current flowing through the ChR2 channels is carried by Na^+^ ions, these channels are also permeable to H^+^ and Ca^2+^ ions^5, 38–40^. Thus, opening of ChR2 channels could produce significant proton and Ca^2+^ influxes, causing intracellular acidification and/or Ca^2+^ overload as reported in glial cells^24^ and oocytes^39^. In cardiac cells, intracellular acidification and Ca^2+^ overload can induce mitochondrial injury and activate Ca^2+^-dependent proteases, leading to cell damage and death^41–44^. However, this mechanism should not be the main cause responsible for the cell toxicity found in the present study because all NRVM cultures were kept in the dark most of the time whereas the signs of cell damage were noticeable from the start of experiments even before optical pacing.

Another possible mechanism underlying the cell injury may be related to the potential deleterious effects of ChR2 overexpression on the function of cell membrane. Previous studies in neurons and other types of cells have shown that overexpression of microbial opsins could alter the morphology and capacitance of the cell membrane, causing membrane damage and cell malformation^25, 45–47^. The resultant cell membrane dysfunction and cytotoxicity eventually led to cell death *via* a variety of signaling pathways including apoptosis. The considerable ChR2 protein aggregation, which was observed in some of the transduced NRVM cultures (Fig. 1A and D), could also contribute to the cell injury. It has been reported that the aggregation of halorhodopsin protein in the endoplasmic reticulum of neuron cells led to bleb formation, local swelling, and cell toxicity^25, 45–48^. The precise contribution of this mechanism to NRVM cytotoxicity could be explored in the future by reducing ChR2 aggregation *via* fusing an ER export signal peptide and/or plasma membrane-targeting signal peptide to the ChR2 sequence as suggested previously^49^.

An important aspect of the present work is the combination of optical stimulation delivered locally *via* a small optical fiber at one location with optical mapping of activation spread in a region spatially separated from the optical pacing site. A possible obstacle of optical mapping is that the light used for excitation of V_m_-sensitive dye RH-237 fluorescence (in the wavelength range of 538–582 nm) could potentially cause partial activation of ChR2, membrane depolarization, and, therefore, change of electrophysiological properties at the mapping site. However, ChR2 absorption spectrum (peak absorption at 470 nm) has only a minor overlap with this wavelength range. Importantly, all measured electrophysiological parameters including conduction velocity, APD_80_ and upstroke rate of rise were similar in ChR2-expressing and control cultures. These data indicate that the effect of the excitation light on ChR2 activity was likely to be small and that optical mapping with RH-237 dye can be combined with optical pacing in cardiac rhythm studies. A definitive proof of this conclusion, however, requires verification using multielectrode electrical mapping of activation spread.

It should be noted that whereas conduction velocity and APD_80_ values measured in control and ChR2-expressing NRVM cultures were comparable to those ones measured previously in isotropic NRVM cultures^50, 51^, the dV/dt_max_ values were slightly lower than previously reported. This difference was likely caused by the lower optical resolution and stronger digital signal filtration (0.2 kHZ) used in the present study. We also showed that optogenetic pacing could be readily expanded to multiple sites. Therefore, optical stimulation may potentially achieve precise control of the location, size, shape and even sequence of illumination, making it a promising candidate for the development of novel cardiac resynchronization therapy device that may complement or replace the conventional biventricular electrical pacing^52, 53^.

A limitation of the present study is the inability to measure action potentials directly at the optical pacing site using current optical mapping approach. One reason for this limitation was the leakage of the blue LED light into the optical V_m_ recording channel. A similar light leakage was reported in a recent study^21^, in which V_m_ recordings were interrupted during optical pulses and the missing V_m_ signal portions were obtained by interpolation allowing examination of the effects of light pulses on APD. This light leakage could be eliminated by using the epi-fluorescent configuration for stimulation light path and better optical filtration (Figure S8). However, V_m_ measurements at the optical pacing site were still precluded due to excitation of the voltage-sensitive dye (RH-237) fluorescence by the blue LED light. Another limitation is the lack of functional analysis of the damaged ChR2-expressing cells such as patch clamp recordings of ionic currents and Ca^2+^ imaging, which could provide insights into more detailed mechanisms underlying the ChR2 expressing-mediated cytotoxicity (i.e. ChR2 functional expression or ChR2 overexpression). This is subject for future studies.

In summary, we developed and characterized a Doxy-inducible optogenetic NRVM culture model that allows direct optical control of electrical cardiomyocyte activity and simultaneous optical mapping of action potential propagation. This study expanded the utility of optogenetic tools in cardiac electrophysiological research.

## Methods

All experimental protocols were approved by the University of Alabama at Birmingham institutional animal care and use committee and adhered to the National Institutes of Health’s guide for the care and use of laboratory animals (NIH Publication No. 85-23, revised 2011).

### ChR2 lentivirus plasmid construction and virus production

The ChR2(H134R)-eYFP insert was amplified from pAAV-CaMKIIa-hChR2(H134R)-eYFP plasmid (Addgene plasmid #26969) using the following PCR primers: GACGAATTCACCATGGACTATGGCGGCGCTTTGTC (forward) and GCGGAATTCTTTACTTGTACAGCTCGTCCATGCC (reverse). The eYFP insert was amplified from the same vector using the following PCR primers: GACGACGAATTCCACCATGGTGAGCAAGGGCG (forward) and GCGGAATTCTTTACTTGTACAGCTCGTCCATGCC (reverse). The TetO-FUW backbone, obtained from the EcoRI-digested TetO-FUW-OSKM (Addgene plasmid #20321), was ligated with EcoRI-digested ChR2-eYFP or eYFP PCR product to generate the inducible lentivirus plasmid TetO-FUW-ChR2-eYFP or TetO-FUW-eYFP (Figure S1A), respectively. The obtained plasmids were sequenced at University of Alabama at Birmingham Helflin Center for Genomic Science to confirm the constructions. To produce lentiviral particles, the TetO-FUW-ChR2-eYFP (or TetO-FUW-eYFP), psPAX2 (Addgene plasmid #12260) and pMD2.G (Addgene plasmid #12259) plasmids were co-transfected to 293T cells using calcium phosphate as described previously^54^. The packed replication-incompetent lentiviral particles were collected, purified, and concentrated on day 5 and stored at −80 °C properly.

The biological titration of lentivirus was performed with HeLa cells. Briefly, around 2.5 × 10^5^ cells were plated in a 12-well plate. On day 2, cell number was counted from one of the wells. TetO-fuw-ChR2-eYFP or TetO-fuw-eYFP virus was thawed and diluted 10-fold serially ranging from 10^−1^ to 10^−4^. Each transduction was performed in duplicate in the presence of 8 μg/ml polybrene. Medium was replaced with addition of 2 μg/ml doxycycline 24 hours after the transduction. Cells were cultured for another two days and then trypsinzed from the plate. The trypsinzed cells were fixed with 1% formaldehyde in phosphate buffered saline (PBS) for 5 minutes, washed with PBS, and then resuspended with 150 μl PBS. eYPF expression were analyzed by FACS at University of Alabama at Birmingham CFAR flow cytometry core. The 1% to 20% eYFP positive dilution was used for titer calculation using the following formula: Number of target cells × (% of eYFP- positive cell/100)/volume of supernatant (in ml).

### NRVM culture production and viral infection

NRVMs were obtained from 1–2 day old neonatal Sprague-Dawley rat hearts as previously described^55, 56^ with some modifications. In brief, hearts from 8–10 rats were excised, the ventricles minced and tissue pieces dissociated in Ca^2+^/Mg^2+^ free Hank’s balanced salt solution that contains 0.1% trypsin and 12 μg/mL pancreatin. The dispersed cells were re-suspended in Ultraculture serum-free medium (Fisher Scientific) supplemented with 2 mM L-glutamine, 2 μg/L vitamin B_12_, 20 U/mL penicillin and 20 μg/mL streptomycin. To reduce fibroblast content, cells were preplated in a 150-cm^2^ culture flask for 2 hours. After preplating, cells were re-suspended in the culture medium and counted. Approximately 2 × 10^5^ cells were plated on collagen-coated cover glasses (diameter 15 mm) and incubated at 37 °C in a humidified atmosphere containing 5% CO_2_ with or without 0.1 mM of bromodeoxyuridine (BrdU).

Two days after cell plating, cultures were transduced using lentivirus containing ChR2-eYFP or eYFP gene with different MOIs (e.g., 10, 20, 40 or 80) in the presence of 8 μg/ml polybrene. Doxycycline (2 μg/ml) was added to the culture 24 hours after the infection to induce transgene expression. The expression of ChR2-eYFP or eYFP was monitored daily with a confocal microscope, which usually reached the peak level in the next 3–4 days and did not decrease significantly within the next week. The efficiency of lentiviral transduction (i.e. percentage of ChR2 positive cells in an infected monolayer) was calculated by dividing the number of YFP-expressing cells by the total number of NRVMs (with and without YFP expression), which were determined from fluorescent and phase contrast images, respectively. The mean fluorescence per monolayer was calculated by dividing the total fluorescence by the total number of cells. Five images were taken for each monolayer, and counts of cells were averaged within the same monolayer for statistical analysis. NRVM cultures were kept in dark and used for optogenetic studies 4–6 days after viral infection.

### Immunocytochemistry

NRVMs cultured in 24-well plates were rinsed twice with PBS and fixed with 3% paraformaldehyde for 20 min at room temperature (RT). Permeabilization was performed by incubating the cultures with 0.1% Triton^TM^X-100 in PBS. After being blocked with 10% inactivated goat serum and 1% bovine serum albumin (BSA) in PBS, the preparations were incubated overnight at 4 °C with an anti-vimentin primary antibody (1:200, mouse monoclonal) or anti-sarcomeric-alpha-actinin primary antibody (1:800, mouse monoclonal). Thereafter, cultures were washed with 0.1% BSA in PBS, blocked again with blocking buffer (30 min at RT), and incubated with an Alexa Fluor 633 secondary antibody (1:800, goat anti-mouse), followed by washing and mounting. Images were acquired using a confocal microscope.

### Cell death assay

The cell viability and cytotoxicity were quantitatively assessed using the trypan blue assay. Briefly, NRVMs in a 24 well plate were washed with PBS twice and incubated at 37 °C, 5% CO_2_ with 200 μL of 0.25% Trypsin-EDTA for 2 minutes. After trypsinization, 800 μL of cell culture medium containing 10% FBS was added to each monolayer. Cell suspensions were harvested and transferred to 15 mL tubes, and diluted with appropriated amount of culture medium. 10 μL cell suspension was then mixed with 10 μL 0.4% trypan blue solution for cell counting. Four areas of hemocytometer were counted and cell numbers were calculated according to the dilution factor and the total medium volume of each monolayer. Cells were counted three times by two individuals independently.

### Confocal imaging of ChR2 expression and recording of light-induced cell contractions

A glass coverslip with NRVM culture was fitted in a perfusion chamber mounted on the stage of a confocal microscope. Cells were perfused with warm (36–37 °C) Hank’s solution. The 515-nm argon laser line of the confocal microscope was used to image YFP distribution. For optical stimulation, a 10x objective lens was coupled to a high-power 470-nm light emitting diode (LED) *via* an optical fiber (diameter 1000 µm) and positioned above the cell monolayer. Optical pulses with duration of 10–20 ms were delivered at a frequency varying between 0.5 and 3.1 Hz using a MyoPacer field stimulator. Cultures were illuminated with white light and changes in transmitted light intensity were video-recorded continuously at 30 fps using a CCD camera mounted on the confocal microscope. Images were analyzed offline using ImageJ software to determine light impulse-induced cell contractions, which were normalized to the displacement of a spot on the cell during spontaneous contraction.

### Simultaneous optical pacing and activation mapping

NRVM cultures were transferred to a recording chamber mounted on a Zeiss Axiovert inverted microscope and continuously perfused with warm Hank’s solution (36–37 °C). For electrical pacing, stimulation pulses were delivered using a bipolar electrode. For optical pacing, an optical fiber (diameter = 1000 µm) coupled to the 470-nm LED was positioned at a distance of ~1 mm from a cell monolayer and 20-ms light pulses were delivered at a rate of 2 Hz. At this distance, the measured diameter of the light spot was ~1.8 mm. The pacing light intensity was calculated as the light power (measured at the fiber exit using an optical power meter) divided by the area of the light spot. The threshold light intensity required for cell pacing was in the range of 1.0–1.3 mW/mm^2^ and the estimated energy was about 50 μJ. To map activation spread, cell cultures were stained with V_m_-sensitive dye RH-237 (2.5 μmol/L) for 5 min. Fluorescence was excited using an Hg/Xe arc lamp and a 560/55-nm excitation filter and measured at >650 nm using a 16 × 16 photodiode array at spatial resolution of 110 µm per diode, as previously described^56^. The mapping area was located at a distance of 2–3 mm from the optical pacing site. The schematic diagram of the optical setup is shown in supplemental Figure S1B.

Optical signals were digitally filtered to increase the signal-to-noise ratio. Activation times were measured at the 50% level of action potential amplitude (APA) and used to construct the isochronal maps of activation spread. Conduction velocity was calculated at each recording site from local activation times and averaged across the whole map using custom-written data analysis software. The maximal upstroke rate of rise (dV/dt_max_) was obtained by taking the first derivative of the optical signal that had been normalized assuming APA value of 100 mV. To measure the action potential duration (APD), an electro-mechanical uncoupler blebbistatin (2.5 μmol/L) was administered to eliminate the motion artifact in some cell cultures. APD_80_ was calculated as the interval between the activation and repolarization time measured at 80% level of the APA.

### Materials

Plasmids for lentiviral vector construction were obtained from Addgene (Cambridge, MA). PCR primers were synthesized by Integrated DNA Technologies (Coralville, Iowa). The qPCR lentivirus titration kit was purchased from Applied Biological Material (Atlanta, GA). The PBS, Triton^TM^X-100, voltage-sensitive fluorescent dye RH-237, and Alexa Fluor 633 secondary antibody were purchased from Life Technologies (Carlsbad, CA). The anti-vimentin antibody, the anti-sarcomeric-α-actinin antibody, and all other reagents were from Sigma-Aldrich (St. Louis, MO).

Data were analyzed using OriginLab software. Comparisons between groups were performed using unpaired 2-tailed Student’s t-test (Figs 1, S3 and S7 and Table 1) or One-Way or Two-Way ANOVA test (Figs 2, S2 and S4). Data were considered significantly different at *p* < 0.05. Results are presented as bar (mean ± SEM) or box chart.

## Electronic supplementary material

Supplementary Information

Supplemental Video
