# Supplementary material for: Electrophysiological Properties and Viability of Neonatal Rat Ventricular Myocyte Cultures with Inducible ChR2 Expression
Source: Sci Rep. 2017 May 8;7:1531. doi: 10.1038/s41598-017-01723-2 (PMC5431527; doi:10.1038/s41598-017-01723-2)
Supplement: Supplementary file 1 — Supplementary Information [file 41598_2017_1723_MOESM1_ESM.pdf]

Online Supplemental Materials

Electrophysiological Properties and Viability of Neonatal Rat Ventricular Myocyte Cultures with Inducible ChR2 Expression

Qince Li[1,4], Rong (Ruby) Ni[1,4], Huixian Hong[4], Kah Yong Goh[1,4], Michael Rossi[2], Vladimir G. Fast[2,3,*], and Lufang Zhou[1,2,3,4,*]

Division of Cardiovascular Disease, Department of Medicine[1], Department of Biomedical Engineering[2], Cardiac Rhythm Management Laboratory[3], and Comprehensive Cardiovascular Center[4], University of Alabama at Birmingham, Birmingham, Alabama, USA

Figure S1. A): Scheme of the constructed doxycycline-inducible lentiviral expression vectors for ChR2 (up) and YFP (lower), and B): Schematic diagram of optical pacing and optical mapping systems. LED-light emitting diode; LS-fluorescent light source; S-shutter; ExF-excitation filter; EmF-emission filter; DM-dichroic mirror; PDA-photodiode array.

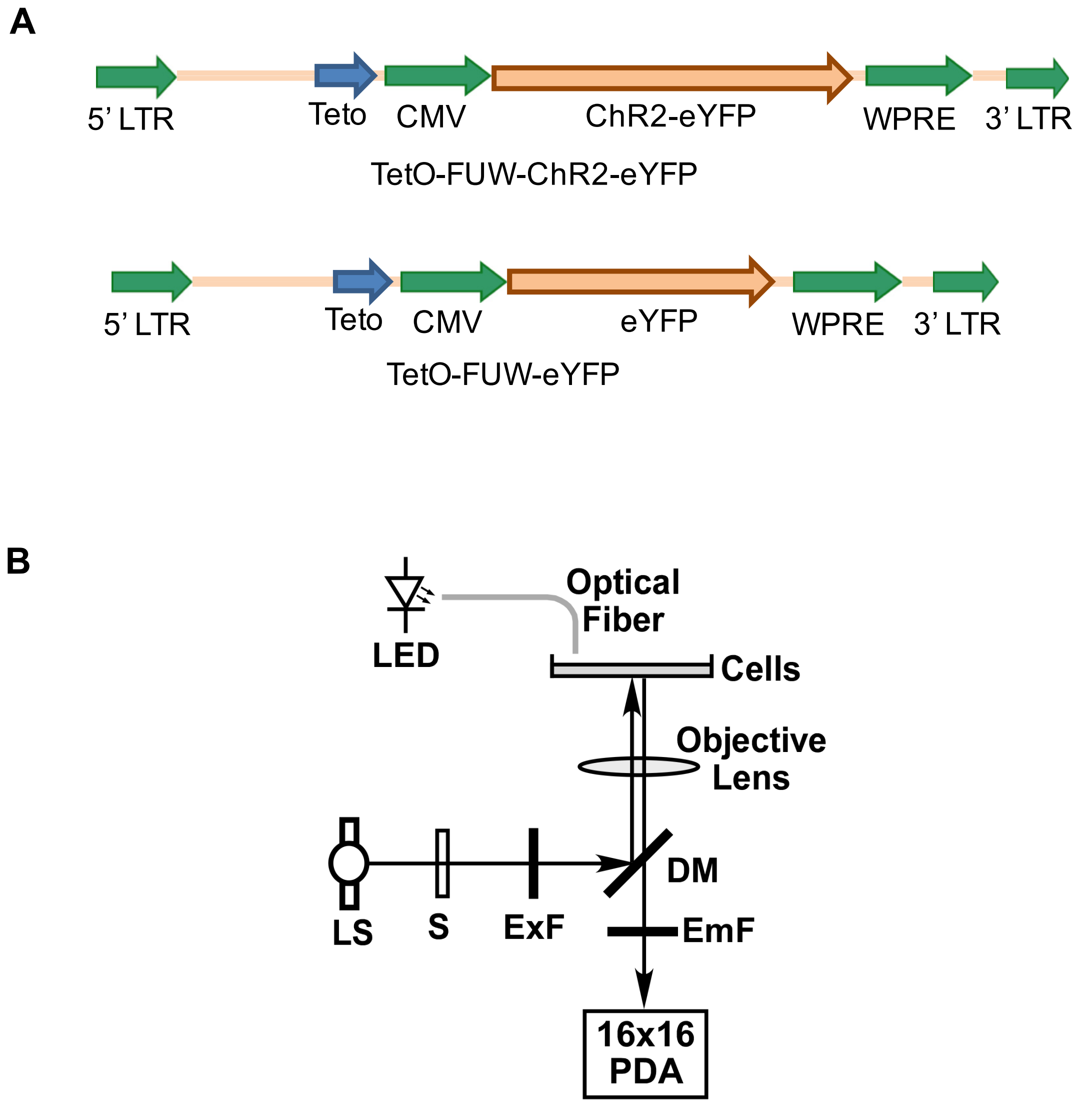

Figure S2. Effects of MOI and BrdU treatment on NRVM monolayers infected with batch #2 ChR2 lentivirus. A): without the treatment of BrdU, and B): with the treatment of BrdU. One-way ANOVA, n=4 for each group.

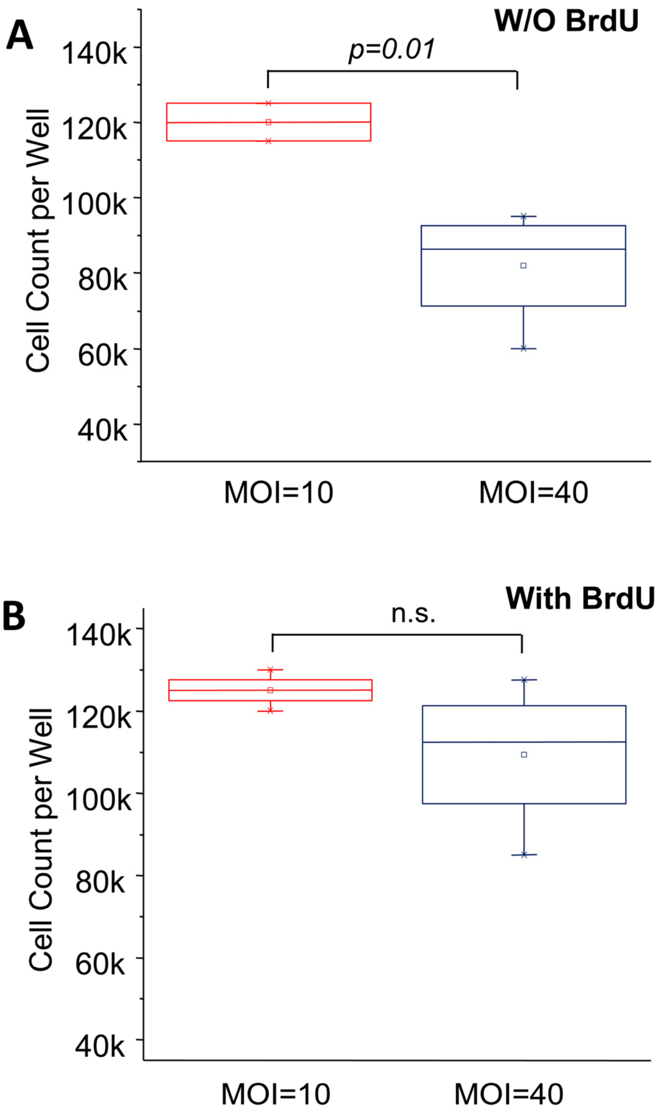

Figure S3. Effect of BrdU or Doxy treatment on the non-infected NRVM cultures. Two-way ANOVA, n=4 for each group.

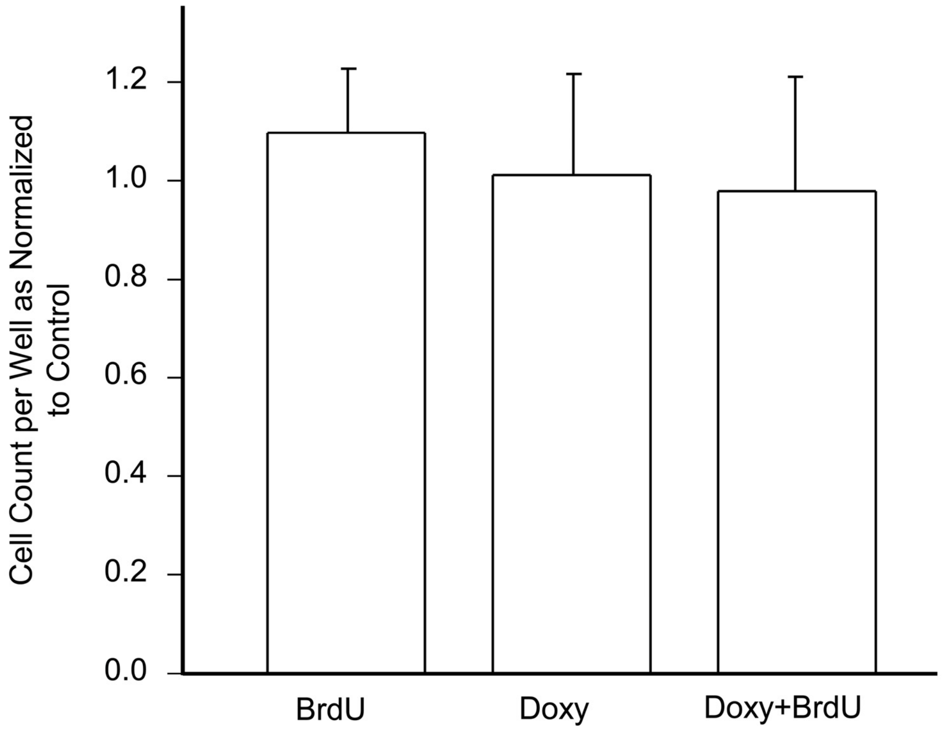

Figure S4. Comparison of cell viability among NRVM cultures infected with low (i.e. 10) or high (i.e. 40) MOI YFP lentivirus, ChR2 batch #1 lentivirus, and batch #2 ChR2 lentivirus. (A) No BrdU treatment, MOI=10; (B) With BrdU treatment, MOI=10, (C) No BrdU treatment, MOI=40, (D) With BrdU treatment, MOI=40. Two-way ANOVA, n=3 or 4 for each group.

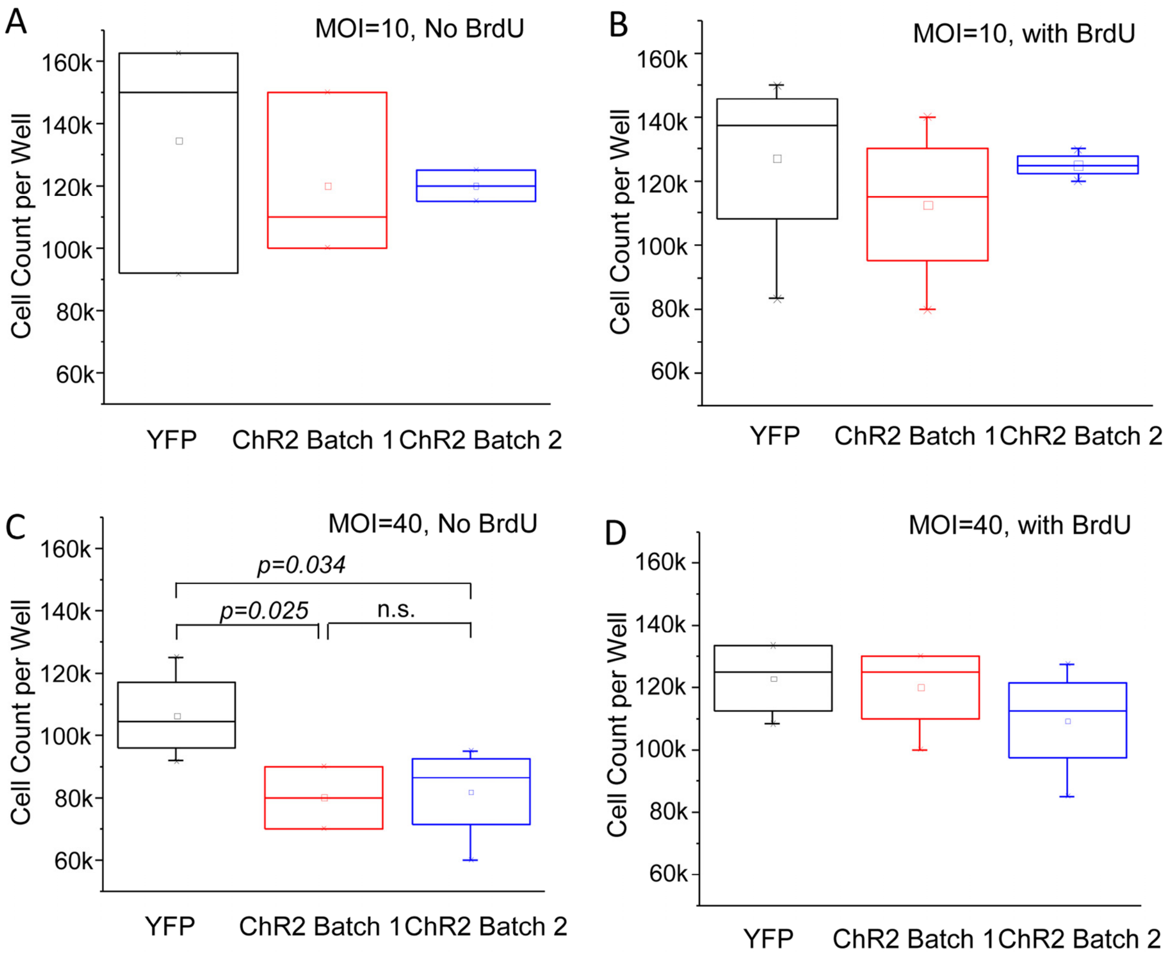

Figure S5. Effect of blebbistatin on control NRVM cultures during electrical pacing. A and B: activation map and optical V_m recordings before blebbistatin treatment; C and D: activation map and optical V_m recordings after blebbistatin application. Blebbistatin suppressed motion artifact without altering the overall activation pattern and the conduction velocity.

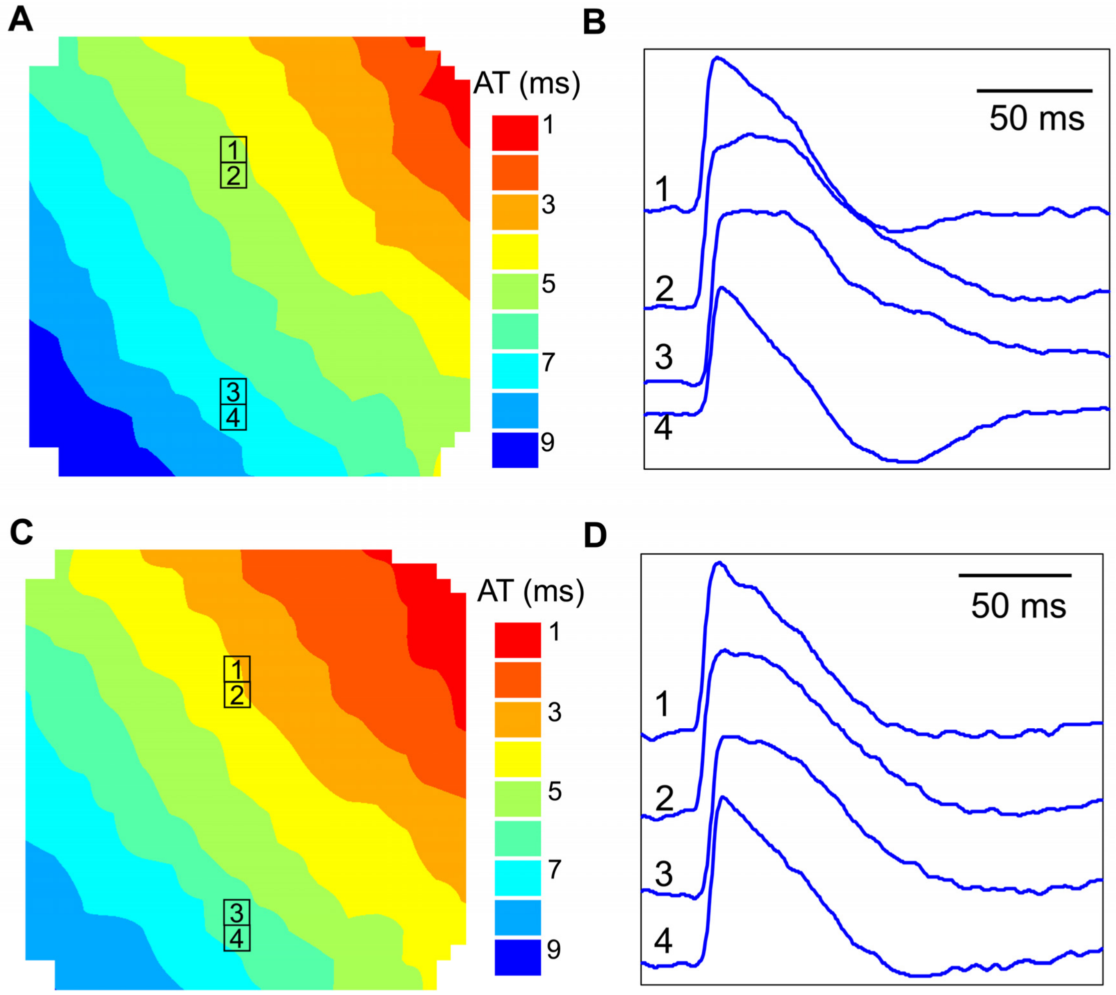

Figure S6. Effect of blebbistatin on ChR2-expressing NRVM cultures during electrical pacing. A and B: activation map and optical V_m recordings before blebbistatin treatment; C and D: activation map and optical V_m recordings after blebbistatin application. Blebbistatin suppressed motion artifact without altering the overall activation pattern.

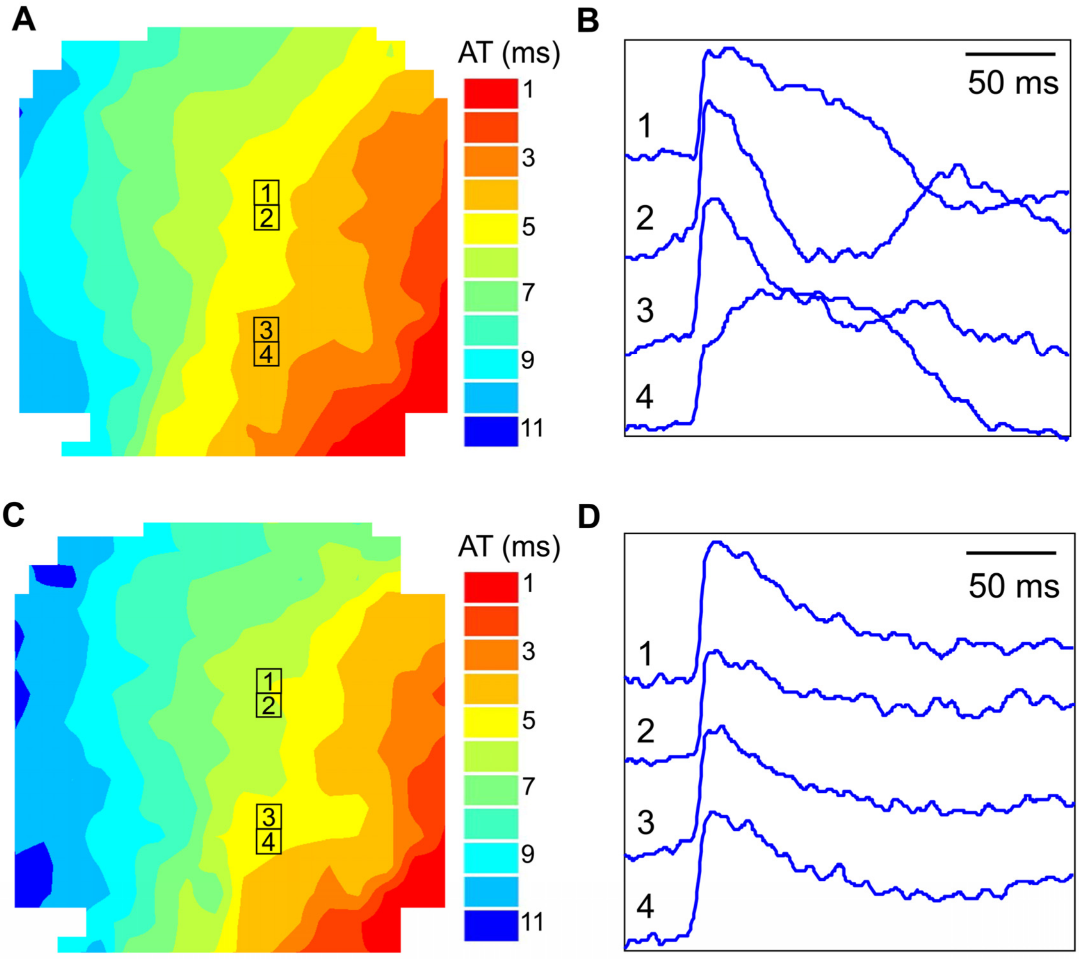

Figure S7. Effect of blebbistatin on conduction velocity in electrical-paced control (A) and ChR2-expressing (B) NRVM cultures, as well as in optical-paced ChR2-expressing cultures (C). There were no significant differences in all groups before and after blebbistatin treatment (two-tailed paired Student's t test). Pacing frequency = 2 Hz.

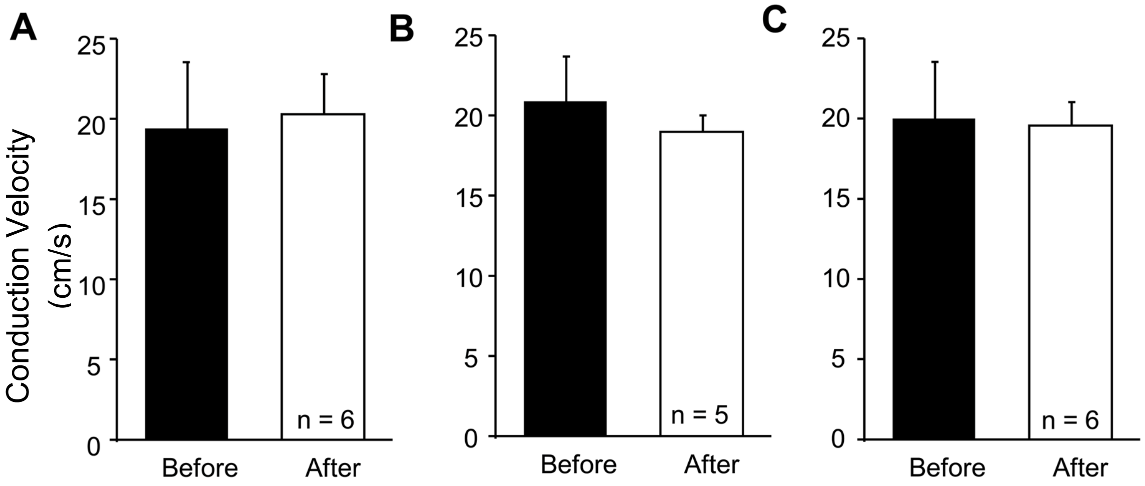

Figure S8. Schematic diagram of proposed illumination and mapping systems that could eliminate the leakage of the blue LED light. (A) The existing LED illuminator. (B) A proposed illuminator for optical stimulation using an epi-fluorescent design. LED light (blue) will be collected with optical fiber OF_1, passed through filter F using two relay lenses, collected by second optical fiber OF_2 and passed through a pinhole P. The pinhole image will be projected onto cells using additional collimator lens C and the objective lens. Changing pinhole diameter will allow to vary the illumination spot size. The holder for the pinhole and the collector lenses will be mounted on an x-y micro-positioner to change the location of the illumination spot within the field of view. Two dichroic mirrors, DM_1 and DM_2, will be used to deflect the stimulation and excitation (green) light toward cells and pass the emitted fluorescent light (red) toward the photodiode array (PDA). LS - fluorescent light source; S - shutter; ExF - excitation filter; EmF - emission filter.

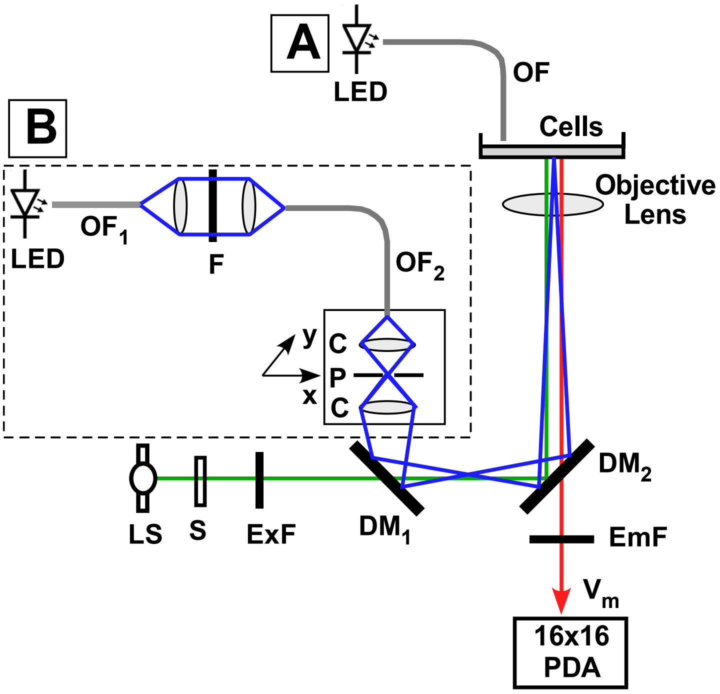

Movie S1. Beating of an NRVM culture paced optically at different frequencies.
